# HER2-positive breast cancer and tyrosine kinase inhibitors: the time is now

**DOI:** 10.1038/s41523-021-00265-1

**Published:** 2021-05-20

**Authors:** Ilana Schlam, Sandra M. Swain

**Affiliations:** 1 MedStar Washington Hospital Center, Washington, DC USA; 2 Georgetown University Medical Center and MedStar Health, Washington, DC USA

**Keywords:** Breast cancer, Targeted therapies

## Abstract

Human epidermal growth factor receptor 2 (HER2) positive breast cancer accounts for 20–25% of all breast cancers. Multiple HER2-targeted therapies have been developed over the last few years, including the tyrosine kinase inhibitors (TKI) lapatinib, neratinib, tucatinib, and pyrotinib. These drugs target HER2 and other receptors of the epidermal growth factor receptor family, therefore each has unique efficacy and adverse event profile. HER2-directed TKIs have been studied in the early stage and advanced settings and have shown promising responses. There is increasing interest in utilizing these drugs in combination with chemotherapy and /or other HER2-directed agents in patients with central nervous system involvement, TKIs have shown to be effective in this setting for which treatment options have been previously limited and the prognosis remains poor. The aim of this review is to summarize currently approved TKIs for HER2+ breast, key clinical trials, and their use in current clinical practice.

## Introduction

Breast cancer (BC) remains the most common type of cancer in women and the second leading cause of cancer related deaths in the United States (US)^1^. Over two million new cases of BC were diagnosed in 2018 worldwide according to the World Health Organization^2^. It is estimated that 276,480 women will be diagnosed with BC in the US in 2020^1,3^. Human epidermal growth factor receptor 2 (HER2+; ErbB2/neu) BC accounts for 20–25% of all BCs; around 360,000 new cases in the world and 48,000 in the US yearly^1,3^.

BC that overexpress HER2 are aggressive and associated with poor prognosis^4,5^. The development of HER2 directed therapies has revolutionized the treatment paradigm and the outcomes of patients with HER2 + BC. Multiple HER2 targeted agents have been approved over the past 5 years for BC, including several tyrosine kinase inhibitors (TKI). In this review we summarize the currently approved TKI for HER2 + BC, key clinical trials, and their use in current clinical practice.

## HER2-positive breast cancer

HER2 is an oncoprotein located in the long arm of chromosome 17^6,7^. HER2 is part of the epidermal growth factor receptor family, which is composed by epidermal growth factor receptor (EGFR)/HER1, HER2, HER3, and HER4 and controls cell growth, survival differentiation, and migration^6–8^.

**Fig. 1:**
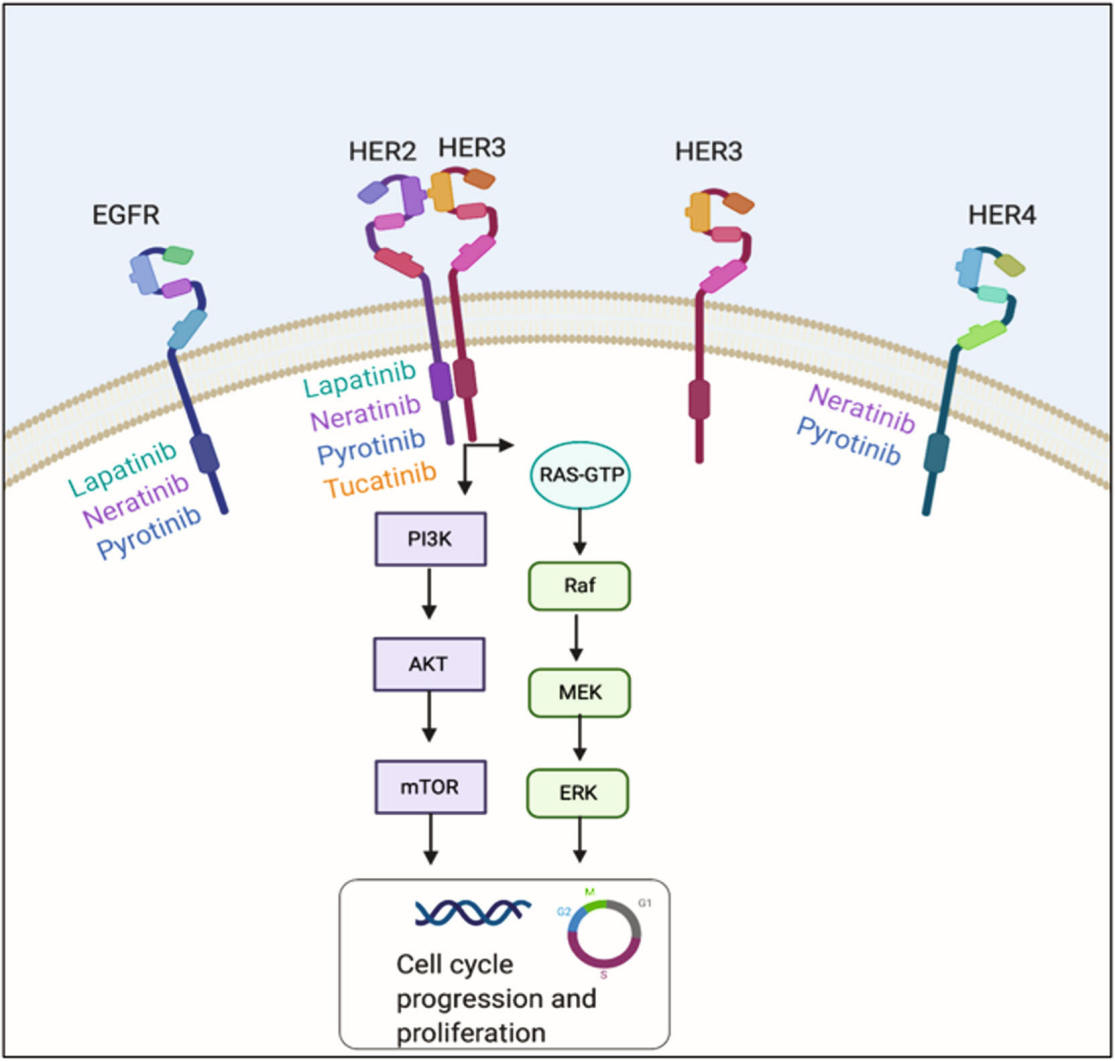
Mechanism of action of HER2 tyrosine kinase inhibitors. There are four members of the HER2 receptor family, these are the targets of lapatinib, neratinib, pyrotinib and tucatinib. The HER2 extracellular domain has no known ligand and is activated by the formation of homo or heterodimers (exemplified by a HER2-HER3 heterodimer in the figure). These dimers lead to phosphorylation of tyrosine kinase residues in the cytoplasmic domain which function as docking sites for proteins that activate the PI3K and MAPK signaling pathways downstream leading to cell cycle progression and proliferation.

The HER2 extracellular domain has no known ligand and is activated by the formation of homo or heterodimers^6,7^. These dimers lead to phosphorylation of tyrosine kinase residues in the cytoplasmic domain which function as docking sites for proteins that activate the phosphatidylinositol triphosphate kinase (PI3K) and mitogen-activated protein kinase (MAPK) signaling pathways (Fig. 1), leading to cell cycle progression and proliferation^6^. Overexpression and amplification are the most common HER2 abnormalities in BC, detected by immunohistochemistry (IHC) or fluorescence in-situ hybridization (FISH), respectively^9,10^.

## HER2-directed therapies

Trastuzumab was approved in the late 1990’s and it was the first HER2 directed therapy approved for the treatment of BC^11^. Since then, several HER2 targeted therapies have been approved including pertuzumab which is another HER2 directed monoclonal antibody and the antibody-drug conjugates ado-trastuzumab emtansine and fam-trastuzumab deruxtecan. Margetuximab, a HER2-directed antibody designed to alter Fc-receptor affinity to CD16 and induce CD16-mediated cytotoxicity was also recently approved^12^.

Small molecule TKIs represent another option for patients with early stage or advanced HER2 + BC. Afatinib is an irreversible inhibitor of EGFR, HER2, and HER4 approved for the treatment of lung cancer with EGFR mutations^13^. Afatinib was one of the first TKIs studied in BC and showed promising results in phase 1–2 studies but failed to demonstrate efficacy in phase 3 trials^13–17^. Lapatinib, neratinib, pyrotinib, and tucatinib are small molecule TKIs that have shown good outcomes in phase 3 trials for patients with HER2 + BC^18–20^. These TKIs have shown to be effective as monotherapy or in combination with chemotherapy, and other HER2 directed agents in the early stage and metastatic settings^18–23^. Studies have shown that patients who progress on trastuzumab may benefit from a HER2 directed TKI with or without trastuzumab^19,20^. This efficacy may be due to different mechanisms of action and target within the HER2 receptor which allows the TKI to overcome mechanisms of resistance to trastuzumab^24,25^.

## Early stage breast cancer

### Lapatinib

Lapatinib ditosylate (GW572016, Kykerb® GlaxoSmithKline) is an orally bioavailable reversible small molecule inhibitor of EGFR and HER2. Lapatinib blocks phosphorylation of the tyrosine kinase residues inhibiting cell proliferation by blocking the MAPK and PIK3 pathways^26^. Preclinical studies demonstrated increased apoptosis in HER2 and EGFR dependent cells in vitro and in vivo^26^. The main pharmacologic characteristics of lapatinib are described in Table 1.

**Table 1:** Characteristics of HER2-targeted tyrosine kinase inhibitors.

	Lapatinib^50^	Neratinib^88^	Pyrotinib^19^	Tucatinib^69^
Inhibition	Reversible	Irreversible	Irreversible	Reversible
Target(s)	EGFR (HER1), HER2 (ErbB2)	EGFR (HER1), HER2 (ErbB2), HER4	EGFR (HER1), HER2 (ErbB2), HER4	HER2 (ErbB2) » EGFR (HER1)
Absorption	Variable (take 1 h before meals)	Increased by 70–120% by high fat meals (>50%)	Variable	Variable
Metabolism	Hepatic, via CYP3A4 and 3A5 > C19 and 2C8	Hepatic, via CYP3A4	Hepatic, via CYP3A4	Hepatic, via CYP2C8 > 3A4
IC50	EGFR 11 nM HER2 9 nM	EGFR 92 nM HER2 59 nM	EGFR 5.6 nM HER2 8.1 nM	EGFR 449 nM HER2 6.9 nM
Excretion	Feces > urine	Feces » >urine	Feces	Feces » urine
Recommended dose	1250 mg PO daily with capecitabine; 1500 mg PO daily with letrozole	240 mg PO daily	400 mg PO daily	300 mg PO twice daily
US FDA approval in the adjuvant setting	✗	✓	✗	✗
US FDA approval in the metastatic setting	✓	✓	✗	✓

*IC50* half maximal inhibitory concentration, *nM* nanomole, *EGFR* epidermal growth factor receptor, *mg* milligrams, *PO* oral administration, *US FDA* United States Food and Drug Administration.

NeoALTTO, GeparQuinto GBG 44, NSABP B-41, and CALBG 40601 were four large phase 3, randomized trials that studied the role of lapatinib in the neoadjuvant setting (summarized in Table 2)^27–30^. In the CALBG 40601 study, patients were randomized to receive paclitaxel plus lapatinib and trastuzumab (THL) or paclitaxel plus lapatinib (TL) or paclitaxel plus trastuzumab (TH) in the neoadjuvant setting. After 7 years of follow up, there was an improvement in recurrence-free survival (THL 93%, TH 79% and TL 69%; hazard ratio 0.32, 95% CI 0.14–0.71, *p* = 0.005) and overall survival (OS) (THL 96%, TH 88%, TL 84%; hazard ratio 0.34, 95% CI 0.12–0.94, *p* = 0.37) in patients that received paclitaxel in combination with lapatinib and trastuzumab when compared with patients that received paclitaxel with one of the HER2 targeted agents (the TL arm was closed early). This was the first time the HER2-enriched BC intrinsic subtype was found to be predictive of response to lapatinib^31–34^. Lapatinib is not currently approved in the neoadjuvant setting and is unlikely to be approved based on published studies since newer TKIs appear to be more effective. However, the biomarker data being generated from the many neoadjuvant trials will be very informative for future research.

**Table 2:** Key phase 3 clinical trials investigating HER-2-targeted tyrosine kinase inhibitors in breast cancer.

Trial information	Study design	Sample size	Population	Findings
**Lapatinib**				
Early stage				
NeoALTTO (Baselga^27^)	Neoadjuvant (+ taxane) and post-neoadjuvant lapatinib (L) vs trastuzumab vs lapatinib (TL) + trastuzumab (T)	455	Women, HER2+, >2 cm localized BC	pCR 51.3% of TL vs 29.5% with T (*p* = 0.0001)
GeparQuinto GBG 44 (Untch^30^)	Neoadjuvant EC followed by docetaxel with trastuzumab (T) vs lapatinib (L)	620	Women, HER2+, lymph node+, BC	pCR 30.3% in T and 22.7% in L (0.04)
NSABP B-41 (Robidoux^29^)	Neoadjuvant AC followed by WT + lapatinib (L) vs trastuzumab vs lapatinib (TL) + trastuzumab (T)	519	HER2+, >2 cm localized BC, LVEF > 50%	pCR 52.5% of T, 53.3% of L, 62% of TL (*p* = 0.95)
CALBG 40601 (Carey^28^)	Neoadjuvant paclitaxel + lapatinib + trastuzumab (THL) vs paclitaxel + lapatinib (TL) vs paclitaxel + trstuzumab	305	HER2+, >1 cm localized BC, LVEF > 50%	pCR 56% TPL, 46% in TP (*p* = 0.13). After 7 years of follow up there was an improvement in recurrence free survival and OS with the triplet^31^
TEACH (Goss^35^)	Adjuvant chemotherapy followed by lapatinib vs placebo	3147	Stage I-IIIc BC, not previously treated with trastuzumab	DFS 13% lapatinib vs 17% placebo (*p* = 0.53; no difference in OS or TTR)
ALTTO (Piccart-Gebhart^36^)^a^	Adjuvant trastuzumab + lapatinib (TL) vs sequential trastuzumab followed lapatinib (T → L) vs trastuzumab alone (T)	8381	HER2+, >1 cm localized BC	No difference in disease free survival (*p* = 0.61) with the addition of lapatinib when compared with T alone
Metastatic				
Lapatinib plus capecitabine (Geyer^46^)	Lapatinib + capecitabine (LC) vs capecitabine (C)	324	HER2+, metastatic BC, who progressed with chemotherapy and trastuzumab	TTP was 8.4 months in LC and 4.4 in C
Lapatinib plus letrozole (Johnston^47^)	Lapatinib + letrozole (LL) vs letrozole + placebo (LP)	219	HR+, HER2+ or −metastatic BC	HER2+: PFS was 8.2 in LL vs 3 months LP
COMPLETE (MA.31) (Gelmon^48^)	Lapatinib + paclitaxel vs trastuzumab + capecitabine	537	HER2+, metastatic BC	PFS was 9 months in the lapatinib arm and 13.6 with trastuzumab (HR 1.48, *p* = .001)
CEREBEL (Pivot^65^)^a^	Lapatinib + capecitabine (LC) vs trastuzumab + capecitabine (TC)	540	HER2+, metastatic BC, no known history of brain metastases	No difference of brain metastases as first site of relapse (*p* = 0.360). OS and PFS longer in the TC arm
**Neratinib**				
Early stage				
ExteNET (Chan^39^)	Extended adjuvant therapy with neratinib (N) for a year vs placebo (P)	2840	HER2+, stage I–III BC that completed therapy including 1 year of trastuzumab therapy	DFS was lower in the N group (*p* = 0.0091). At 8 years follow up there was a an absolute benefit of 7.4% in iDFS and 9.1% in OS for patients who initiated treatment <1 year post-trastuzumab^40^
Metastatic				
NEfERT-T (Awada^50^)	Neratinib + paclitaxel (NP) vs trastuzumab + paclitaxel (TP)	480^b^	Previously untreated HER2+ metastatic BC	Median PFS was 12.9 months with NP and 12.9 months with TP. The incidence of CNS metastases was lower in the NP arm (relative risk 0.48, *p* = 0.004)
NALA (Saura^20^)	Lapatinib + capecitabine (LC) vs neratinib + capecitabine (NC)	621	HER2 + , metastatic BC, received two lines of HER2-directed therapy	PFS was improved with NC (*p* = 0.0059). No difference in OS. DOR was 8.5 months in NC and 5.6 in LC (*p* = 0.0004). Fewer interventions to CNS disease in NC vs LC (*p* = 0.043)
**Tucatinib**				
Metastatic				
HER2CLIMB (Murthy^19^)	Tucatinib + trastuzumab + capecitabine (TTC) vs placebo + trastuzumab + capecitabine (PTC)	612	HER2 + , metastatic BC, received two lines of HER2-directed therapy^c^	PFS at 1 year was 33.1% in TTC vs 12.3% in PTC (*p* = 0.001). PFS was better in TTC 7.8 vs 5.6 months. OS in TTC was 44.9 vs 26.6% in PTC (*p* = 0.005)

*pCR* pathologic complete response, *EC* epirubicin, cyclophosphamide, *AC* doxorubicin, cyclophosphamide, *WT* weekly taxane, *LVEF* left ventricular ejection fraction, *DFS* disease-free survival, *OS* overall survival, *TTR* time to first recurrence, *PFS* progression-free survival, *TTP* time to progression, *HR* hormone receptor, *CNS* central nervous system, *DOR* duration of response.

^a^Terminated early.

^b^Initially designed as a phase 3 study but accrual was reduced from 1200 to 480, therefore it was no longer powered as a phase 3 study and results were considered preliminary according to the conclusions of the authors.

^c^PFS, OS, and intracranial ORR was better in patients that received TTC vs PTC.

Lapatinib has also been studied in the adjuvant setting (selected studies summarized in Table 2). The TEACH study randomized patients who received neoadjuvant chemotherapy without trastuzumab to receive lapatinib or placebo. There was no difference in disease free survival (DFS) between the arms^35^. In the ALTTO study, patients were randomized to four treatment groups: trastuzumab (T) with or without chemotherapy, lapatinib (L) with chemotherapy, sequential treatment with T for 12 weeks followed by L or concurrent therapy T + L. Higher rates of DFS were noted in the combination arms; however, none were statistically significant and patients treated with lapatinib had higher rates of diarrhea, rash, and hepatic toxicity^36^. Lapatinib was associated with increased toxicity with a limited improvement in outcomes, therefore this drug is not currently used in the neoadjuvant or adjuvant setting.

### Neratinib

Neratinib (HKI-272, Nerlynx®, Puma Biotechnology) is an irreversible inhibitor of EGFR, HER2, and HER4^37^. Preclinical studies demonstrated that neratinib leads to cell cycle arrest and decreased proliferation of HER2 + cells^37^. The key pharmacologic characteristics of neratinib are described in Table 1.

The I-SPY 2 (NCT01042379) trial is an adaptive phase 2 trial that is evaluating targeted agents in combination with standard chemotherapy in the neoadjuvant setting. In this study, patients received paclitaxel in combination with neratinib followed by doxorubicin and cyclophosphamide. Patients in the control group received trastuzumab in combination with paclitaxel, again followed by doxorubicin and cyclophosphamide. This study included 52% hormone receptor (HR) positive tumors and 57% HER2+. A pathologic complete response (pCR) was achieved in 56% of patients in the neratinib group vs 33% in the control group in patients with HER2+/HR– BC. For HER2+/HR + disease the pCR rates were 30 and 17%, respectively^38^.

The exteNET study included patients with stage 2–3 HER2 + BC that had completed neoadjuvant or adjuvant trastuzumab in combination with chemotherapy. A total of 2840 women were randomized to receive adjuvant neratinib or matching placebo for 12 months^39^. Neratinib provided greater benefit in invasive-free survival to patients with HR + disease (hazard ratio 0.51, 95% CI 0.33–0.77, *p* = 0.0013) than to those with HR- BC (hazard ratio 0.93, CI 0.60–1.43, *p* = 0.74)^39^. At 8 years of follow up, in all patients neratinib was associated with an improvement in OS with an absolute benefit of 2.1% and an absolute benefit in invasive DFS of 9.1%^40^. The patients that received the TKI had lower rates of central nervous system (CNS) events^40^. Patients in this study did not receive pertuzumab or TDM1, both currently used as standard of care in early stage BC, therefore the degree of benefit of neratinib in tumors pretreated with these medications remains unknown.

### Current approach

Neratinib is the only TKI currently approved for early-stage BC in the US. This drug was approved in 2017 as extended adjuvant anti-HER2 therapy after completion of trastuzumab, based on the exteNET trial^39^. Neratinib is often used in patients with HR + and HER2 + high-risk tumors however, its use is often limited due to high incidence of diarrhea. Antidiarrheal prophylaxis and dose escalation have been shown to decrease the incidence and severity of this toxicity^41^.

There has been a trend in BC to utilize more agents in the early stage setting to decrease the incidence of recurrences and progression to metastatic disease. There are several ongoing trials to assess other combinations that include TKIs in the neoadjuvant and adjuvant settings (Table 3). Many of these studies are incorporating TKI in the initial adjuvant or neoadjuvant therapy rather than in the extended adjuvant setting. Based on the impressive findings of the HER2CLIMB study, there is particular interest in the use of tucatinib. Another arm of the described ISPY2 trial (NCT01042379) is ongoing to assess the combination of tucatinib with standard chemotherapy in the neoadjuvant setting and the compassHER-RD study (NCT04457596) is evaluating the use of ado-trastuzumab-emtansine with or without tucatinib in patients with residual disease after neoadjuvant therapy^42,43^.

**Table 3:** Ongoing key phase 2–3 trials investigating HER-2-targeted tyrosine kinase inhibitors in breast cancer.

Trial information	Description (sample size)	Endpoints
Early stage		
NCT01042379 (I-SPY 2) phase 2	Neoadjuvant tucatinib + trastuzumab + pertuzumab + paclitaxel → doxorubicin + cyclophosphamide	pCR RBC, relapse free survival, OS, safety
NCT03101748 phase 1b/2	Neoadjuvant neratinib + paclitaxel + trastuzumab + pertuzumab → doxorubicin + cyclophosphamide vs neratinib + paclitaxel → doxorubicin + cyclophosphamide (*n* = 99)	pCR Maximum tolerated dose of neratinib, PFS, safety
NCT04457596 (compassHER RD) phase 3	Adjuvant tucatinib + ado-trastuzumab emtansine vs ado-trastuzumab emtansine in patients with residual disease after neoadjuvant therapy (*n* = 1031)	Invasive DFS distant DFS, CNS metastases DFS, OS
NCT03085368 phase 3	Adjuvant epirubicin + cyclophosphamide → docetaxel + lapatinib vs epirubicin + docetaxel + lapatinib vs epirubicin + cyclophosphamide → docetaxel + trastuzumab vs epirubicin + docetaxel + trastuzumab (*n* = 482)	DFS OS
Advanced/metastatic disease		
NCT01670877 phase 2	Neratinib in HER2 mutant (not amplified) mBC or neratinib + fulvestrant in HR +/HER2 mutant mBC (*n* = 80)	ORR PFS, safety
NCT03975647 (HER2CLIMB02) phase 3	Tucatinib + ado-trastuzumab emtansine vs placebo + ado-trastuzumab emtansine vs placebo (*n* = 460)	PFS OS, PFS per RECIST, ORR, DOR, clinical benefit, adverse events
NCT04539938 (HER2CLIMB04) phase 2	Tucatinib + ado-trastuzumab deruxtecan (*n* = 70)	ORR DOR, PFS, disease control rate, OS, adverse events
NCT03054363 phase 1b/2	Tucatinib + palbociclib + letrozole in ER + /HER2 + mBC (*n* = 25)	Tolerability PFS
Advanced/metastatic disease with CNS involvement		
NCT01494662 phase 2	HER2 + mBC and CNS metastases: neratinib vs neratinib + surgical resection vs capecitabine (in patients previously treated with/without lapatinib) vs neratinib + ado-trastuzumab emtansine (*n* = 168)	ORR PFS, OS, CNS response, site of progression, safety and tolerability, clinical outcomes
NCT04334330 phase 2	HR + HER2 + mBC with CNS metastases: palbociclib, fulvestrant, trastuzumab, and lapatinib (*n* = 48)	CNS response rate Safety, OS, PFS, ORR, time to CNS progression, time to radiotherapy
NCT03501979 phase 2	HER2 + mBC with leptomeningeal disease: tucatinib + trastuzumab + capecitabine (*n* = 30)	OS Safety, PFS, CNS ORR, clinical benefit, symptom burden, quality of life
NCT04512261 (TOPAZ) phase 1/2	HER2 + mBC with CNS metastases: tucatinib in combination with pembrolizumab and trastuzumab (*n* = 33)	CNS disease control rate. Recommended dose of tucatinib. CNS ORR, systemic ORR, PFS, OS, toxicity profile

*pCR* pathologic complete response, *RCB* residual cancer burden; *PFS* progression-free survival, *OS* overall survival, *ORR* objective response rate, *TTP* time to progression, *DOR* duration of response, *CNS* central nervous system, *mBC* metastatic BC, *ER* hormone receptor.

## Advanced breast cancer

### Lapatinib

Lapatinib was initially studied in the metastatic setting in phase 1 and 2 trials^44,45^. In a phase 2 study the use of lapatinib in patients with advanced HER2 + BC resulted in objective response rate (ORR) of 24%, the median time to response (TTR) was 7.9 months, and the median duration of response (DOR) was 28.4 weeks. Four patients developed asymptomatic reductions in ejection fraction (EF)^45^. These findings lead to development of several phase 3 clinical trials.

A phase 3 trial compared capecitabine plus or minus lapatinib in patients with locally advanced or metastatic and previously treated HER2 + BC. In the combination arm, the median time to progression was 8.8 months vs 4.4 months with capecitabine alone (hazard ratio 0.49, 95% CI 0.34–0.71, *p* < 0.001). Similarly, the PFS was 8.8 months vs 4.1 months, respectively (hazard ratio 0.47, 95% CI 0.33–0.67, *p* < 0.001). Eleven patients in the capecitabine arm developed brain metastases vs four in the combination arm (*p* = 0.10)^46^. Johnston et al. compared lapatinib plus letrozole vs letrozole alone in postmenopausal women with locally advanced or metastatic HR + BC. In women with endocrine receptor and HER2 + disease, the addition of lapatinib improved PFS when compared with letrozole alone, 8.2 months vs 3 months, respectively (hazard ratio 0.71, *p* = 0.019)^47^. The COMPLETE (MA.31) study compared HER2 directed therapy with lapatinib vs trastuzumab plus paclitaxel for 24 weeks followed by the same HER2 directed agent until disease progression in women with HER2 + metastatic BC. The PFS was 9.1 months in the lapatinib arm and 13.6 months with trastuzumab (hazard ratio 1.48, *p* > 0.001)^48^. Lapatinib is currently approved by the United States Food and Drug Administration (US FDA) for advanced or metastatic HER2 + BC in combination with capecitabine or with letrozole for patients that received prior therapy including an anthracycline, a taxane, and trastuzumab^49^.

### Neratinib

NEfERT-T was initially a phase 3 study but accrual was reduced from 1200 to 480 therefore it was no longer powered as a phase 3 study and results were considered preliminary according to the conclusions of the authors^50^. In this trial, women with previously untreated metastatic HER2 + BC were randomized to receive neratinib plus paclitaxel vs trastuzumab and paclitaxel. The median PFS was 12.9 months in both groups. The ORR, clinical benefit rate and DOR were similar for both groups^50^. The phase 3 NALA trial compared lapatinib plus capecitabine vs neratinib plus capecitabine in patients with previously treated metastatic HER2 + BC with at least two prior lines of therapy. PFS was improved in the neratinib arm (hazard ratio 0.76, 95% CI = −0.63–0.93, *p* = 0.0059). There was no statistically significant difference in OS. The neratinib arm also had higher ORR and DOR. Patients with HR- disease had the greatest benefit from this combination (HR- hazard ratio 0.76, 95% CI 0.57–1.01 and for HR + hazard ratio 0.94, 95% CI 0.72–1.22). In contrast with the exteNET trial in which patients with HR + disease had a greater benefit from this TKI in the extended adjuvant setting (DFS HR + hazard ratio 0.51 vs 0.93 for HR-)^20,39^. Based on these studies, neratinib is approved by the US FDA in combination with capecitabine for patients who received at least two lines of HER2 directed therapy.

### Tucatinib

Tucatinib (ONT-380, Tukysa®, Seattle Genetics) is a highly selective reversible HER2 inhibitor^51^. Tucatinib showed efficacy in preclinical models as monotherapy and in combination with chemotherapy and trastuzumab^51^. Additionally, xenograft models showed that BC were more sensitive to tucatinib than to lapatinib, the response to tucatinib was improved with the addition of trastuzumab^52^. The key pharmacologic characteristics of tucatinib are described in Table 1.

Tucatinib was approved by the US FDA in April of 2020 for the treatment of HER2 + metastatic BC, based on the phase 3 HER2CLIMB study. This was the first drug approved under Project Orbis, a collaboration between the US FDA and other international regulators to allow uniform access to therapies and clinical trials globally. In HER2CLIMB, patients with metastatic HER2 + BC who were previously treated with trastuzumab, pertuzumab, and ado-trastuzumab emtansine were randomized in a 2:1 ratio to receive trastuzumab, capecitabine, and tucatinib vs trastuzumab, capecitabine plus placebo. The PFS at 1 year was 33.1% for the tucatinib arm and 12.3% for the placebo arm (hazard ratio for disease progression or death was 0.54, 95% CI 0.42–0.71, *p* = <0.001). The OS at 2 years was 44.9% in the tucatinib arm and 26.6% in the placebo arm. The median PFS was 7.8 months and 5.6 months, respectively^19^. A recent update of the HER2CLIMB trial revealed that the benefit of tucatinib in PFS occurred irrespective of HR status^53^. HER2CLIMB included patients that received trastuzumab, pertuzumab, and ado-trastuzumab emtansine. However, the trial did not specify the setting. If a patient developed metastatic disease while receiving adjuvant trastuzumab and pertuzumab and was given ado-trastuzumab emtansine as first line treatment, at the time of progression the patient would have been eligible for HER2CLIMB. The HER2CLIMB regimen can be considered for patients with metastatic BC who received at least one HER2-targeted based regimen.

There is growing interest in the combination of tucatinib with antibody drug conjugates. A phase 1b trial by Borger et al.^54^ studied the combination of ado-trastuzumab emtansine and tucatinib in 57 patients with heavily pretreated metastatic BC; the ORR was 48% and the median PFS was 8.2 months. There are ongoing trials phase 3 trials assessing safety and efficacy of antibody drug conjugates and tucatinib in the metastatic setting (summarized in Table 3).

### Pyrotinib

Pyrotinib is an irreversible inhibitor of EGFR, HER2, and HER4. This TKI was approved in China in 2018 in combination with capecitabine for patients with advanced or metastatic HER2 + BC. A phase 2 study by Ma et al.^18^ compared lapatinib plus capecitabine to pyrotinib plus capecitabine. The median PFS was 18 months in the pyrotinib arm and 7 months in the lapatinib arm and the response rate was 78.7% and 57.1% respectively. The most common side effects were hand foot syndrome, diarrhea, and neutropenia^18^. In the phase 3 study by Jiang et al.^55^, pyrotinib plus capecitabine was compared to placebo plus capecitabine in women with metastatic BC. The median PFS was 11 months in the pyrotinib arm and 4.1 months in the placebo arm. Interestingly, patients that progressed on the placebo arm were then treated with pyrotinib monotherapy and had a single agent response rate of 38% with a median PFS of 5.5 months^55^. The PHOEBE trial compared pyrotinib plus capecitabine vs lapatinib vs capecitabine in 267 patients with metastatic HER2 + BC. The median PFS was significantly longer in the pyrotinib arm (12.5 months) than in the lapatinib arm (6.8 months, *p* < 0.0001). Diarrhea and hand foot syndrome were more common in the pyrotinib arm^56^. There are multiple ongoing trials assessing the use of pyrotinib in different settings, it remains unclear if its use will expand to other countries.

### Brain metastases

Brain metastases occur in up to 50% of patients with HER2 + BC and the development of central nervous system (CNS) metastases is associated with dismal outcomes^57,58^. The use of systemic therapy for brain metastases has been historically limited due to low CNS penetrance and limited efficacy of these medications^59–61^. Recently, a subgroup analysis of the KAMILLA study, which assessed the efficacy of ado-trastuzumab emtansine in metastatic HER2 + BC, revealed that 42.9% of the patients with baseline measurable brain metastases (*n* = 126) had a reduction in the size of the CNS lesions by at least 30%^61^. Of the patients with baseline brain metastases that did not undergo radiation therapy, 49.3% of the 67 had an at least 30% reduction of the size of their brain metastases^61^. Although systemic treatments for HER2 + BC brain metastases are improving, there is still an unmet need for better and durable therapies for this group of patients and TKIs represent a promising option for these patients^57,58^.

Lapatinib has shown to have CNS penetrance. In a phase 2 study of 242 patients with treated HER + BC brain metastases, CNS ORR was observed in 20% of patients treated with capecitabine and lapatinib^62^. Similarly, in the single arm phase 2 LANDSCAPE trial the combination of lapatinib and capecitabine in 45 patients with untreated BC brain metastases, reported 65.9% of the patients had an intracranial response, all partial responses^63^. A retrospective subgroup analysis of the EMILIA trial (which compared capecitabine plus lapatinib vs ado-trastuzumab emtansine) showed similar PFS for both arms in patients with baseline BC brain metastases. However, the PFS was 9.6 months in the ado-trastuzumab emtansine arm and 6.4 months in the capecitabine plus lapatinib arm (hazard ratio 0.65; *P* < 0.001). In patients with CNS disease there was a significant improvement in OS in the ado-trastuzumab emtansine arm (26.8 vs 12.9 months, hazard ratio 0.38 *p* = 0.008)^64^. In the phase 3 CEREBEL trial 540 patients with metastatic HER2 + BC were randomized to receive capecitabine plus trastuzumab vs capecitabine plus lapatinib. The incidence of brain metastases as first site of relapse was 3% in the lapatinib group and 5% in the trastuzumab group (*p* = 0.360). PFS and OS were longer in the trastuzumab arm and serious adverse events were more commonly reported in the lapatinib arm^65^.

Neratinib has also been studied in patients with brain metastases. The phase 2 study TBCRC 022 assessed the combination of neratinib and capecitabine in 49 participants with BC brain metastases with or without prior lapatinib. Patients were eligible if they had at least one lesion measuring 10 mm or more and with CNS progression after prior local therapies. The CNS ORR was 49% in participants not previously treated with lapatinib and 33% in patients who previously received this medication. The median PFS was 5.5 and 3.1 months, respectively^66^. In the NEfERT-T study (neratinib plus paclitaxel vs trastuzumab and paclitaxel) symptomatic or progressive CNS disease occurred in 8.3% patients in the neratinib arm and 17.3% of the trastuzumab arm (*p* = 0.002), the cumulative incidence of brain metastases was 20.2% in the neratinib arm and 10.1% in the trastuzumab arm (*p* = 0.002). Of note, twice as many patients had baseline CNS involvement in the trastuzumab arm compared to the neratinib arm, these patients were diagnosed with symptoms prior to the study as NrfERT-T did not include baseline screening for brain metastases^50^. The phase 3 NALA trial included patients with stable or asymptomatic brain metastases. This study showed improved PFS and time to CNS intervention with neratinib-capecitabine when compared with lapatinib-capecitabine and included participants with stable and asymptomatic brain metastases. In NALA, 22.8% of patients on the neratinib arm and 29.2% on the lapatinib arm required CNS-directed interventions (*p* = 0.043)^20^. These studies have shown CNS penetration and clinical efficacy of neratinib in pretreated patients with HER2 + BC brain metastases which may be improved compared to lapatinib.

Tucatinib in combination with trastuzumab and capecitabine showed impressive results in the HER2CLIMB trial. This study was unique in that it included 291 patients with brain metastases (*n* = 198 in the tucatinib arm and *n* = 93 in the placebo arm), which were classified as active or stable based on a baseline magnetic resonance imaging. Forty percent of the patients had treated or stable CNS disease, 37% treated and progressing, and 22% untreated brain metastases. The risk of intracranial progression or death was reduced by 68% in the tucatinib arm. The intracranial ORR was 47.3% in the tucatinib arm vs 20% in the control arm (*p* = 0.03). The median central nervous system (CNS) PFS was 9.9 months vs 4.2 months and the median PFS was 18 months vs 12 months, respectively. In the subset of patients whom did not receive radiation therapy, the CNS PFS was 8.1 months in the tucatinib arm vs 3.1 months in the control arm. The 1-year OS in the tucatinib arm was 70.1 vs 46.7% in the control arm. CNS progression occurred in 21 patients in the tucatinib arm and nine in the control arm; these patients underwent local therapy and then continued their study treatment. In those patients the time from random assignment to second progression was 15.9 months in the tucatinib arm and 9.7 months in the control arm (*p* = 0.009) and the median time from progression in the brain to second progression or death was 7.6 and 3.1 months, respectively^67^. Tucatinib was the first FDA approval to specifically include patients with brain metastases in the indication statement. Based on these results, there has been growing interest to use tucatinib in the first line metastatic setting for patients with BC brain metastases^68^. The ongoing TOPAZ trial (NCT04512261) is assessing the combination of tucatinib with the immune checkpoint inhibitor pembrolizumab and trastuzumab in patients with HER2 + BC brain metastases.

### HER2 mutations

Although amplification and overexpression are the most commonly present alterations in HER2, activating mutations have been described as an alternative driver mechanism and a poor prognostic factor in BC^9,10^. HER2 mutations are found in 2.8% of BCs. Though not routinely performed, next generation sequencing use is increasing in the metastatic setting, and its use will likely continue to grow as more targeted agents are developed and approved^9,10^.

Neratinib has been studied in HER2 mutant tumors in the phase 2 SUMMIT trial^69^. Forty-six patients with HR+ and HER2 mutant metastatic BC received the combination of neratinib, trastuzumab, and fulvestrant. In this cohort, 12 allelic variations of HER2 were identified with L755S accounting for 33%. Clinical activity was evaluated in 30 out of the 46 patients and 12 (40%) achieved a partial response and none achieved a complete response. The median DOR was 8.4 months. And the median PFS was 8.3 months^70^. Other TKIs are being studied in this setting^9^.

### Mechanisms of resistance to HER2-directed therapies

Intrinsic and acquired mechanisms of resistance to HER2-targeted agents have been described. HER2 reactivation or signaling downstream from the HER2 receptor are some of the commonly described mechanisms of resistance. HER2 mutations are a well-known intrinsic and acquired mechanisms of resistance^69,71^. Different types of mutations have been described, including activating mutations and mutations that modify the receptor preventing binding of monoclonal antibodies^25,71,72^. L755S is the most frequently identified acquired activating mutation of HER2 which is more commonly seen in metastatic tumors. L755S has been described as an acquired mechanism of resistance to lapatinib and cross resistance to tucatinib has been reported in vitro. However, the resistant cells appear to be sensitive to neratinib, which is a pan-HER2 TKI^71,73^. More studies are needed to determine the clinical significance of this mutations to allow us to tailor treatment based on the specific mutations.

Another known mechanism of resistance is the incomplete blockade of the HER2 family receptor which can lead to upregulation of several HER family receptors^74^. This mechanism can be overcome by adding pertuzumab to trastuzumab or combining it with a potent TKI with the goal of targeting different HER2 family receptors^25^. For example, an increase in HER3 transcription has been described after treatment with lapatinib. This mechanism is mediated by AXL activation. A multi-kinase inhibitor, such as foretinib a MET inhibitor, which has activity against AXL can overcome this mechanism of resistance in vitro^75,76^.

Alterations in downstream signaling pathways have also been described as a resistance mechanism. For example, activating mutations in *PIK3CA* or low levels of expression of tumor suppressor genes (such as PTEN) can lead to tumor proliferation^25,77,78^. The cyclin pathway also plays a role in resistance to HER2-targeted agents. Preclinical data has shown that the sensitivity to HER2-directed therapies can be restored by targeting cyclin dependent kinases 4 and 6^79^. Bidirectional cross talk between HER2 and estrogen receptors is another known resistance mechanism for tumors that express both receptors. As estrogen receptor can serve as an escape pathway to HER2 inhibition, concurrent blockade can prevent this phenomenon in vitro^80^.

The tumor immune infiltrate also plays a key role in the response to trastuzumab. Tumors with higher quantities of tumor infiltrating lymphocytes (TIL) have been associated with a better response to trastuzumab^81–83^. In the MA.31 study, patients with lower levels of cytotoxic stromal TIL had a greater benefit with trastuzumab when compared to lapatinib. Future studies are needed to determine the predictive role of TIL in the response to different HER2 TKI^84^. PDL1 expression has been proposed as a mechanism of resistance to trastuzumab. The combination of HER-targeted agents with immune checkpoint inhibitors has shown to be effective in patients with PDL1 positive metastatic disease who were previously treated with trastuzumab^85,86^. There are multiple studies ongoing to continue to identify mechanisms of resistance and strategies to overcome resistance, as well as the role of TKIs in this setting^25,70^.

### Safety profile

Each TKI targets different HER-receptors therefore these drugs are associated with unique toxicity profiles. Table 4 summarizes the most commonly reported adverse events (AE) based on landmark studies for HER2 + metastatic BC.

### Gastrointestinal toxicities

Although all the HER2 TKIs are associated to diarrhea, neratinib, and pyrotinib use leads to higher rates in all grades (83 and 97% respectively), likely due to effects on EGFR. The use of neratinib requires intensive loperamide prophylaxis and the FDA recommends to start loperamide with the first dose of neratinib and continue for the first two cycles to maintain 1–2 bowel movements a day. This is particularly important to ensure adherence in the extended adjuvant setting^66,87^. The CONTROL trial assessed the use of different regimens of antidiarrheal prophylaxis in patients receiving adjuvant neratinib. Dose escalation was started at 120 mg daily for a week, followed by 160 mg daily for a week then increased to 240 mg daily in one arm and in the other arm it was started at 160 mg daily for 14 days, followed by 200 mg daily for 14 days and then increased to 240 mg daily. There were four regimens for diarrhea prophylaxis that included loperamide, budesonide, and/or colestipol. These interventions, particularly budesonide and loperamide as well as dose escalation, lead to lower incidence, duration, and severity of diarrhea. In the CONTROL trial 3–12% of patients required dose reductions when compared to 26% of the patients in the exteNET trial. All patients receiving neratinib should receive antidiarrheal prophylaxis and dose escalation should be considered^41^. Similarly, tucatinib has been associated with diarrhea in 81% of cases, with only 12% being grade 3 and 0.5% grade 4. Antidiarrheal prophylaxis was not specified in the HER2CLIMB trial, however loperamide and/or budesonide can be utilized as prophylaxis and to treat this common side effect^19,88^. Other common gastrointestinal AE are listed in Table 4.

**Table 4:** Selected adverse events and laboratory abnormalities from phase 2/3 studies.

	Lapatinib^20,46^		Neratinib^20^		Pyrotinib^18^		Tucatinib^19^	
Grade	Any (%)	≥3 (%)	Any (%)	≥3 (%)	Any (%)	≥3 (%)	Any (%)	≥3 (%)
**Gastrointestinal**								
Diarrhea	60	13	83	24	97	15	81	13
Nausea	44	4	53	4	39	2	58	4
Vomiting	26	2	46	4	62	5	36	3
Stomatitis	15	0	21	2	11	0	26	3
Anorexia	15	1	35	3	32	2	25	1
Increased AST	51	–	6	1	26	3	21	5
Increased ALT	42	–	10	1	29	2	20	5
**Mucocutaneus**								
PPE syndrome	49	7	46	10	79	26	63	13
Rash	27	1	10	0	5	2	20	–
**Cardiovascular**								
Decreased LVEF	2	1	4	0	–	–	–	–
**Other**								
Headache	9	0	11	0	–	–	22	1
Anemia	16	4	15	2	22	2	–	–
Pyrexia	16	1	11	0	9	0	–	–

*AST* aspartate aminotransferase, *ALT* alanine aminotransferase, *PPE* palmar plantar erythrodysesthesia, *LVEF* left ventricular ejection fraction.

### Cardiotoxicity

Cardiac toxicity has been reported in patients taking TKI which is particularly relevant as these medications are often given in combination or after other cardiotoxic treatments, such as anthracyclines, trastuzumab, and/or radiation therapy. Many of the pivotal clinical trials excluded patients with baseline cardiac abnormalities therefore the rates of cardiac toxicity may have been underestimated. Lapatinib was initially associated with a decrease in the left ventricular ejection fraction (LVEF) in 2–5% of patients^49^. Neratinib has not been associated with this toxicity^87^. Tucatinib was associated with a decrease in LVEF in 1% of patients^88^. Given that the incidence of symptomatic decrease in LVEF is low there and there are no clear recommendations for cardiac monitoring, however cardiac imaging (echocardiogram) can be considered in a case by case basis for patients with known heart failure or with multiple cardiovascular risk factors who have received cardiotoxic cancer-directed therapies.

## Conclusions and future directions

**Fig. 2:**
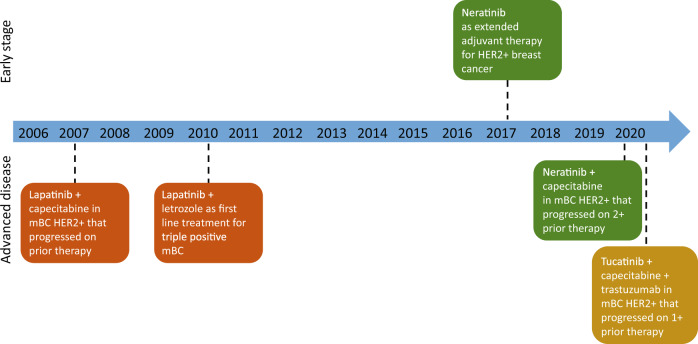
United States Food and Drug Administration and Approval History of HER2-targeted tyrosine kinase inhibitors. This shows the time of approval and indications of the currently tyrosine kinase inhibitors approved by the United States Food and Drug Administration.

HER2-targeted TKIs have been approved for specific populations of BC patients (Fig. 2). With the increasing number of therapies approved for HER2 + BC, there are a lot of unanswered questions; particularly about efficacy and safety of drug combinations and toxicities. Another unanswered question is if there is cross resistance of TKI and how to select TKI based on prior HER2 directed treatments.

**Fig. 3:**
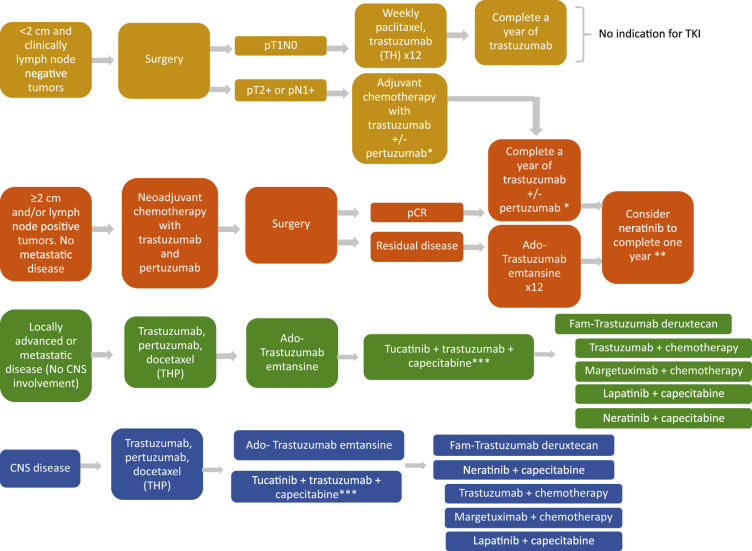
Proposed treatment algorithm for HER2+Breast Cancer. This is a suggested treatment algorithm for early stage and advanced human epidermal growth factor 2 positive BC, based on the current United States Food and Drug Administration approvals. Radiation therapy and endocrine therapy should be incorporated when appropriate. There are multiple options for metastatic disease beyond second line, tucatinib can be considered in the third line setting based on the results of the phase 3 HER2CLIMB study. Other agents can be considered as fourth line or beyond, based on prior therapies, toxicity profile and comorbidities. *Subgroup analysis of APHINITY showed that patients with node positive disease have a greater benefit from adjuvant dual HER2 blockade. **Benefit seen in patients with hormone receptor positive and HER2 positive disease. There are no data about the use of neratinib after treatment with pertuzumab and/or ado-trastuzumab emtansine. ***Tucatinib is FDA approved in the second line setting after treatment with trastuzumab, pertuzumab and ado-trastuzumab emtansine. Can be considered for patients with CNS involvement.

Figure 3 shows the current proposed treatment algorithm based on the US FDA approved HER2-targeted agents. Note that there are multiple options for metastatic disease beyond second line. Tucatinib can be considered in the third line setting based on the results of the phase 3 HER2CLIMB study, particularly in those with CNS involvement^19,67^. Other agents can be considered as fourth line or beyond, based on prior therapies, toxicity profile, and comorbidities. Fam-trastuzumab deruxtecan showed durable responses in heavily pretreated patients with metastatic HER2 + BC in a phase 2 single arm study^89^. We are awaiting for the results of the phase 3 trials to better assess effectiveness and toxicity profile and to determine the optimal line of therapy for this promising antibody-drug conjugate^89^. Margetuximab was recently approved in the metastatic setting for patients that received at least two prior lines of therapy based on the phase 3 SOPHIA study^90^. Other options include the previously mentioned lapatinib and neratinib in combination with capecitabine^20,46,47^. The details about these trials and drugs are outside of the scope of this review.

Does BC phenotype particularly HR status, plays a role in the TKI selection? Lapatinib is the only TKI approved in combination with endocrine therapy, however, 48–60% of patients enrolled in the mentioned landmark phase 3 studies for the other TKIs had HR+ disease and all groups benefited from TKI therapy. A recent update of the HER2CLIMB trial revealed that the benefit of tucatinib was irrespective of HR status^53^. Based on the current information, HR status should not play a significant role while selecting HER2-targeted TKI^19,20,46,47,53^. There is an ongoing study to determine the safety and efficacy of the combination of a HER2 TKI with a cyclin dependent kinase 4 and 6 inhibitor (NCT03054363). Preclinical models suggested that the combination of tucatinib, abemaciclib and fulvestrant lead to significant tumor regressions^52^. However, a clinical trial that attempted to combine abemaciclib with tucatinib and trastuzumab (NCT03846583) was withdrawn. Both tucatinib and abemaciclib are metabolized by CYP3A and tucatinib has shown to inhibit CYP2C8, CYP2C9, and CYP3A which could increase the plasma concentration of both drugs and therefore increase their toxicity making the combination difficult to tolerate^91,92^.

It is possible that tucatinib or other highly selective HER2 TKI will be used more broadly in the near future, as the results from the initial trials were impressive, including CNS efficacy, and it appears to be better tolerated. The COMPASS-RD trial will examine tucatinib added to ado-trastuzumab emtansine in the adjuvant setting. The HER2CLIMB2 study is also assessing the use of ado-trastuzumab emtansine with or without tucatinib in patients with HER2 + metastatic BC. The single arm phase 2 HER2CLIMB4 study (NCT04539938) will assess the use of fam-trastuzumab deruxtecan in combination with tucatinib.

Other HER2 targeted TKIs are being investigated. Epertinib (S-22611) is a reversible inhibitor of EGFR, HER2, and HER4 that has shown good results in combination with trastuzumab with or without capecitabine in a phase 1/2 study that included patients with CNS disease^23^. Varlitinib (ASLAN001) is another reversible inhibitor of EGFR, HER2, and HER4 that is being studied in combination with chemotherapy for advanced solid tumors in the NCT02396108 trial^22^. Poziotinib is an irreversible inhibitor of EGFR, HER2, and HER4 that has also been studied in BC^93^. Ongoing studies will help determine their efficacy and unique toxicity profiles as well as the role of this pan-HER TKI in the treatment of HER + BC.

Some remaining questions include the duration of HER2-directed TKI in patients with early stage BC and for those with metastatic disease that achieve a complete response and a good quality of life. The role of TKI in de-escalation of therapy has also not been fully elucidated. Another remaining question is treatment sequencing, as it is possible that some drugs allow for better responses used as first line. Also, combinations with immunotherapeutic agents are a promising area of research in HER2 positive BC and the role that TKIs play in this arena is unknown.

The treatment paradigm of HER2 + BC is evolving rapidly, leading to improved prognosis and quality of life of patients diagnosed with early and metastatic disease. However, there are still many unanswered questions and there are ongoing trials to try to find the answers to further optimize the treatment and outcomes of this patient population for best quality of life.

## Data Availability

Source data for all figures and tables are provided in the paper. No new data sets have been generated or analyzed for this article.
